# Walloons as General or Specific Others? A Comparison of anti-Walloon and anti-immigrant Attitudes in Flanders

**DOI:** 10.5334/pb.336

**Published:** 2017-11-21

**Authors:** Bart Meuleman, Koen Abts, Cecil Meeusen

**Affiliations:** 1 University of Leuven, BE; 2 Tilburg University, NL

**Keywords:** intergroup relations, anti-Walloon prejudice, anti-immigrant prejudice, group-focused enmity, perceived threat, structural equation modeling

## Abstract

This study attempts to shed light on the structure, the prevalence and the determinants of anti-Walloon attitudes in Flanders. For this purpose, we contrast anti-Walloon prejudice with prejudice against a relatively well-understood and archetypical out-group, namely immigrants. Our theoretical approach draws on insights from two paradigms of intergroup relations: the Group-Focused Enmity approach stressing that specific prejudices have a strong common denominator, and the Differentiated Threat model arguing that specific prejudices are contingent on the context of intergroup relations as well as the involved types of threat. To assess the (dis)similarities in anti-Walloon and anti-immigrant prejudice, we use the Flemish dataset of the Belgian National Election Study (BNES) 2010. Comparable measurement instruments for both forms of prejudice are analyzed by means of structural equation modeling. Our results reveal a nuanced picture regarding the similarities and differences between anti-Walloon and anti-immigrant attitudes in Flanders. One the one hand, anti-Walloon and anti-immigration attitudes are strongly correlated and rooted in economic threat perceptions. On the other hand, anti-Walloon attitudes are less outspoken in the Flemish population than anti-immigrant attitudes, are less founded on cultural threat perceptions and are more closely linked to feelings of identification with the Flemish in-group.

## Introduction

Since the inception of the Belgian nation state, the -at times tense- intergroup relations between Flemish and Francophone citizens have been a staple of political conflict. In spite of six Belgian state reforms, providing more autonomy for the regions and communities, the linguistic/territorial cleavage has not been pacified. While the historical, legal and political dimensions of the linguistic and territorial conflicts in Belgium have received ample scholarly attention (e.g. Deprez & Vos, 1998), relatively few recent studies have investigated intergroup attitudes between Flemings and Francophones or Walloons (for exceptions, see Duriez et al., 2013; Klein et al., 2012). In fact, the analysis of attitudes towards ‘Wallonians’ -i.e. a fictitious ethno-religious minority group invented by Hartley (1946)- had greater resonance in the prejudice literature than empirical research of prejudices towards real-existing Walloons.

Yet, the attitudinal dimension of the regional and communitarian conflicts remains a factor of political importance. Especially in Flanders, the linguistic and territorial conflicts have been politicized during recent elections, and negative imagery regarding the other group has been mobilized (Abts, Swyngedouw & Meuleman, 2015). These strategies of ‘Othering’ appeal to feelings of economic as well as cultural threat among the Flemish public, and make use of stereotypical images such as ‘profiteering unemployed Walloons’, ‘French-speaking bourgeois’ and ‘corrupt Walloon politicians’ (Klein et al., 2012).

The purpose of this contribution is to shed more light on the prejudices that Flemish Belgians hold towards Francophone citizens. Because of the complex federal structure -Belgium is not only divided in three language communities (Dutch-speaking, Francophone, and German-speaking), but also in three regional entities (Flanders, Wallonia, and Brussels bilingual region)- the Flemish are confronted with a subnational ‘Other’ that has two different faces. On the one hand, there is the territorial divide between Flanders and Wallonia that appeals to the existence of different economic and political realities between Flemish and Walloons. On the other hand, the linguistic distinction between Flemish and Francophones evokes symbolic and cultural differences. The economic Other (Walloon) and the cultural Other (Francophone) do not necessarily overlap, as cultural threats are especially salient in the Brussels region and its periphery, where Flemish and Francophones live together. As our results will show, however, the distinction between these two faces of the subnational Other is very blurry in Flemish public opinion. In absence of a more appropriate term that includes both aspects, we will use the term anti-Walloon attitudes throughout this study.^1^

Furthermore, we approach intergroup attitudes in Belgium from a comparative perspective and contrast anti-Walloon attitudes with attitudes towards immigrants. Not only are the origins of anti-immigrant attitudes relatively well-understood (Wagner, Christ & Heitmeyer, 2010), immigrants (in Belgium especially of Turkish and Moroccan descent) can be considered the archetypical out-group to which negative feelings are directed (Meuleman et al., in press; Zick, Pettigrew & Wagner, 2008). Comparing attitudes towards Walloons/Francophones and immigrants thus allows us to address the specificity (vs. generality) of French-speaking Belgians as a target group of negative attitudes.

Concretely, this contribution sets out to answer three research questions, relating to the *structure*, the *level* and the *roots* of anti-Walloon attitudes: (1) Do anti-Walloon and anti-immigrant attitudes show the same structure in terms of sources of threat (symbolic vs. realistic)? (2) Are anti-Walloon and anti-immigrant attitudes equally prevalent in the Flemish population? (3) Are anti-Walloon and anti-immigrant attitudes in similar ways linked to social background characteristics and individual difference variables, such as authoritarianism, relative deprivation, and (sub)national identification? Or are anti-Walloon attitudes -due to the particular position of the French-speaking ‘Other’ in the Belgian context- specific in their structure and genesis? To answer these questions empirically, we analyse data from the Belgian National Election Study (BNES) of 2010 by means of structural equation modelling (SEM).

## Theory and previous research

To inform our comparison of the structure, prevalence and roots of anti-Walloon and anti-immigrant prejudices, we combine insights from two paradigms on intergroup relations (Choma & Hodson, 2008). The Group-Focused Enmity (GFE) approach stresses that prejudices towards various groups are highly similar in structure and origins. The Differentiated Threat (DT) model conversely assumes that prejudices are group-specific and ultimately dependent on the concrete intergroup context (Meuleman et al., in press). Below, we elaborate on the differences between the two approaches, describe the context of the intergroup relations between Flemings and respectively Walloons and immigrants, and derive hypotheses regarding the differences and similarities between anti-Walloon and anti-immigrant prejudice.

### The Syndrome of Group-focused enmity: the common denominator of prejudice

Inspired by Allport’s work on *the Nature of Prejudice* (1954), numerous empirical studies have shown that attitudes towards various out-groups are highly correlated: someone who holds prejudices against one specific out-group, for instance immigrants, is also likely to be relatively prejudiced against other, seemingly unrelated groups, such as homosexuals, Jews, or elderly people (Bergh & Akrami, 2016; Zick et al., 2008). Basically, individuals have a consistent tendency to evaluate all kind of out-groups in a similar vein and categorize them as one ‘Band of Others’ (Kalkan, Layman & Uslaner, 2009). Building on this idea of ‘generalized prejudice’, Zick et al. (2008) show that different types of prejudice cluster into a single syndrome of group-focused enmity (GFE), with at its core an ideology of group-based inequality. This syndrome of GFE has been confirmed across a diversity of target groups and cultures (Bratt, 2005; Davidov et al., 2011; Meeusen & Kern, 2016).

Following the logic of GFE, the tendency to systematically dislike all sorts of out-groups can be explained by individual-difference variables such as personality traits, ideological dispositions and cognitive styles, rather than by group-specific situational factors (Akrami, Ekehammer & Bergh, 2011). Right-wing authoritarianism (RWA) and social dominance orientation (SDO), for example, have been identified as two of the strongest predictors of generalized prejudice (Ekehammar et al., 2004; Van Hiel & Mervielde, 2005). Authoritarians typically adhere to the maintenance of traditional values, an uncritical submission to authorities, and the rejection of norm-violators (Altemeyer, 1998). People high on RWA tend to reject a wide array of out-groups because these groups are perceived to be threatening the social order, the norms and values of the in-group (Duckitt & Sibley, 2010). Similarly, persons with a strong SDO, i.e. the tendency to support hierarchy and inequality between social groups (Pratto et al., 1994), are more inclined to devalue out-groups because of their lower social status. Also the social identity perspective is compatible with the GFE approach: the stronger one identifies with the in-group, the more one is inclined to think in terms of in- vs. out-groups, which fosters feelings of prejudice toward out-groups in general (Tajfel & Turner, 1986). In sum, the existence of GFE implies that the targets of the syndrome are disliked for similar reasons and therefore have mutual origins (Zick et al., 2008).

### Differentiated threats: the out-group specificity of prejudice

Others, however, have criticized the generalized prejudice approach for neglecting the concrete structural positions, competitive contexts and cultural-discursive space in which intergroup relations are embedded (Blumer, 1958; Meuleman et al., in press; Rubin & Hewstone, 2004). As such, the focus on individual difference variables should be complemented by attention for the context of intergroup relations. A central tenet of this situational approach is that out-groups become the target of prejudice because the dominant group feels that these out-groups pose a threat to certain prerogatives of the in-group (Riek, Mania & Gaertner, 2006). The intergroup threat theory makes a clear distinction between realistic and symbolic sources of threat (Stephan, Ybarra & Morrison, 2009). On the one hand, realistic or socio-economic threats are induced by experiences of relative deprivation as a result of competition for scarce resources such as territory, material well-being, political and economic power, but also the provisions of the welfare state (Meuleman, Davidov & Billiet, 2009; Sherif, 1966). Symbolic or cultural threats, on the other hand, originate in intergroup conflict over the established social order, cultural traditions, and shared beliefs, values and norms (Stephan & Stephan, 2000).

Because the type and salience of threat induced are contingent on specific characteristics of the intergroup context, distinct out-groups can evoke differential attitudinal reactions among the majority group members. By consequence, prejudices do not necessarily generalize across target groups. Contrary to the GFE approach, the *differentiated threat model* (DT; Meuleman et al., in press) postulates that idiosyncrasies of the target group and intergroup relations in terms of economic and cultural threat are crucial in defining the structure and origins of prejudice. Out-groups can predominantly be perceived as an economic threat, a cultural threat, or a combination of both. The Dual Process Model of Duckitt and Sibley (2010) stresses that prejudice towards a specific group can have divergent roots, depending on the nature of invoked threat. Concretely, prejudice against out-groups that challenge the in-group’s values and norms is predominantly driven by authoritarian dispositions, as especially individuals with strong beliefs in authority, order and conventions will feel threatened by groups challenging their social-cultural standards. Conversely, individuals who view society in terms of competition for social dominance and power will direct prejudice primarily toward groups challenging their dominant social position. Similarly, negative sentiments towards out-groups competing for scarce economic resources are primarily induced by feelings of group relative deprivation (Pettigrew et al., 2008). Prejudices against groups that are both economically and culturally threatening are driven by SDO, RWA as well as relative deprivation (Asbrock, Sibley & Duckitt, 2010).

### The specificity of anti-Walloon and anti-immigrant attitudes

In sum, while the GFE approach assumes that the structure and origins of prejudice should be similar across group-specific prejudices, the DT model argues that anti-Walloon/Francophone and anti-immigrant prejudices have -at least partially- differentiated structures and roots due to the specific cultural and economic contexts in which the relations between Flemings and these groups are embedded. We thus evaluate two traditional approaches – the individual difference perspective emphasising the generality of prejudice and the differentiated threat perspective accentuating the particularity and context-dependence of group-specific prejudices – in one overall framework. In this section, we contextualize the specific positions of both immigrants and Walloons/Francophones in Belgian society, and explore arguments for the existence of group-specific antecedents of anti-Walloon and anti-immigrant attitudes.

Historically speaking, relations between Flemings and Francophones have been structured by two interrelated conflicts based on symbolic (cultural identity) and realistic (material interests) resources. The first grievances of the Flemish movement were mainly focused on linguistic and cultural issues. Since the onset of mobilization, narratives of past cultural oppression and linguistic discrimination against the Flemish identity have structured the *cultural-linguistic conflict*. Even though the initial power imbalance has shifted from the Francophones to the Flemings, the Flemish historical discourses of past injustices and cultural dominance still inspire nationalist claims (Farhat, Rosoux & Poirier, 2014). Anchored in these historical narratives, the Francophones are depicted as a threat to the cultural unity of the Flemish community. At the level of social stereotypes, the Francophones are perceived as arrogant, contemptuous and feeling superior (Klein et al., 2012: 22–24). Nowadays, the linguistic issue remains especially present in the Brussels periphery where Francophones residing on Flemish territory are blamed for not assimilating to the monolingual language regime (De Winter & Baudewyns, 2009). Outside the Brussels periphery, however, Flemish and French-speaking Belgians largely occupy non-overlapping cultural spaces.

While cultural-linguistic issues have decreased in salience, the *political-economic conflicts* about autonomy for the regions and socio-economic redistribution have gained dominance in current Flemish nationalist discourse. The autonomy claim captures the current core of Flemish grievances towards the Francophones who are not only accused of threatening its cultural heritage, but also slowing down economic dynamism of Flanders and impairing its democratic functioning (Farhat, Rosoux & Poirier, 2014). On the economic axis, the claim is that wealthy Flanders is subsidizing poor Wallonia through the centralized social insurance system (Béland & Lecours, 2005). So, the rivalry with the Walloons involves a competition for resources from the federal welfare state. The Flemish call for increasing autonomy emphasizes the illegitimacy of the financial transfers between Flanders and Wallonia using populist statements of ‘lazy Walloons living on the rents of the hardworking Flemish tax-payers’ and of an ‘inefficient, clientelistic, and even corrupt Walloon government squandering public money’ (De Winter & Baudewyns, 2009: 294). In the political-economic conflict, the stereotypical image of the independent, productive, hard-working and autonomous Fleming is opposed to the dependent, non-productive, lazy and profiteering Walloons (Klein et al., 2012: 24–26).

Compared to Walloons/Francophones, the structural and discursive positions of immigrants in Flanders show similarities as well as differences. Post-war labor migration has attracted immigrants coming from Southern Europe (predominantly Italy) and later also North-Africa and Turkey. Over the course of the years, however, Flemish citizens started to associate the notion of ‘immigrants’ predominantly with the Turkish and Moroccan communities (Spruyt, van der Noll, & Vandenbossche, 2016). This group of immigrants generally occupies disadvantaged socio-economical positions and is often perceived as a threat for low-skilled jobs and social welfare provisions of the native Flemings (Abts & Kochuyt, 2013). Especially regarding the scarce resources of the welfare state, immigrants and Walloons are perceived as posing similar threats. As a culturally visible and distinct out-group, immigrants of Turkish and Moroccan origin are also perceived as a threat to the established social-cultural order (Swyngedouw, 1995). Compared to Walloons, however, the perceived threat to the Flemish norms and values can be expected to be more salient. First, from the Flemish perspective, the cultural differences with Turkish and Moroccan immigrants, who are predominantly Muslims, are more outspoken than those with Francophone Belgians. Second, while there is (apart from the Brussels area) a strong spatial separation between Flemings and Walloons, immigrants are visibly present in and changing the face of Flemish cities, thereby increasing the breeding ground for perceptions of symbolic threat.

Summarizing, Flemings share a single economic space with Walloons as well as immigrants, thereby competing for the same scarce resources of the welfare state. Regarding the cultural dimension, intergroup relations with immigrants and Francophones are no longer symmetrical: while Flemings and immigrants compete for symbolic goods within the same cultural sphere, Flemings and Francohpones largely occupy separated cultural spaces (apart from the Brussels region).

### Similarities and differences in structure, level and roots

Combining these theoretical perspectives (GFE and DT), supplemented with information regarding the context of intergroup relations in Flanders, we develop –sometimes competing- hypotheses regarding the similarities and differences between the structure, prevalence and determinants of anti-Walloon and anti-immigrant attitudes.

First, the *structure of prejudice* refers to its constituent components and the interrelation between these components. The GFE approach postulates that the structure of prejudices is universal within a society, irrespective of the specific target group (Zick et al., 2008). As such, GFE theory hypothesizes anti-Walloon and anti-immigrant attitudes to be structured along the same lines (H1a). The DT approach, however, argues that the content of prejudice is context dependent and that salience of cultural and/or economic threat defines how the prejudice type is structured. Given the specific context of intergroup relations in Flanders, we expect the cultural dimension to be more salient for anti-immigrant prejudice than for anti-Walloon prejudice (H1b).

Second, regarding the *level of prejudice*, Flemings are expected to have more positive attitudes towards Walloons than towards immigrants (H2). After all, the DT approach argues that Flemings perceive a larger cultural distance -and consequently symbolic threat- towards immigrants than towards Walloons. Note that the hypothesized difference in level of anti-Walloon and anti-immigrant attitudes not necessarily contradicts the GFE perspective. The GFE approach predicts that attitudes towards various out-groups are highly correlated, but correlation does not necessarily imply that negative attitudes towards the respective groups are equally negative in a given population (Zick et al., 2008).

Third, the GFE and DT perspectives lead to opposing hypotheses regarding the impact of three key *determinants of prejudice*, namely authoritarianism, relative deprivation and national (vs. subnational) identification. The GFE approach focuses on the common bases of prejudices, and therefore predicts that these determinants will be related in identical ways to various forms of prejudice, irrespective of the specific out-group. Concretely, GFE expects that anti-Walloon and anti-immigrant prejudices are to the same extent induced by identification with the Flemish in-group (H3a), authoritarianism (H4a) and relative deprivation (H5a).

The DT model, conversely, stresses the existence of a unique component of prejudices that is idiosyncratic to the target group and the intergroup context. First, regarding (sub-)national identification, we expect a differential impact. Because Belgian citizens live in a federal state, they can identify with their community (Flemish or Francophone), with the state of Belgium or with both entities. This dual national identity conception has differential consequences for prejudice. From a historical point of view, it has been argued that whereas the dominant discourse of Belgian citizenship is rather civic, i.e. celebrating the values of diversity and tolerance, conceptions of Flemish identity are more ethno-cultural, i.e. based on blood, culture and language (Billiet, Maddens & Beerten, 2003; Duriez et al., 2013). As both immigrants and Walloons may threaten this Flemish cultural heritage, identification with the Flemish in-group is positively related to prejudice toward both groups (Billiet, Maddens & Beerten, 2003). However, historically speaking the Francophones and Walloons have served as a whetstone for the construction of Flemish identity. Because Flemish identity is explicitly positioned against Francophones/Walloons, we expect it to be more strongly related to anti-Walloon attitudes than toward anti-immigrant attitudes (H3b). Second, the DT model also predicts differential effects for authoritarianism. Since both immigrants and Walloons place a strain on the cultural identity of the Flemish, we expect authoritarianism to be positively related to negative anti-immigrant as well as anti-Walloon attitudes. However, because the perceived symbolic threat stemming from Walloons will be lower compared to the cultural threat posed by immigrants, authoritarianism will have a weaker positive effect on anti-Walloon attitudes compared to anti-immigrant attitudes (H4b). Finally, because Walloons and immigrant pose a similar threat to the collective resources of the welfare state in the eyes of the Flemish, the DT model predicts relative deprivation to be equally related to anti-Walloon and anti-immigrant attitudes (similar as H5a derived from the GFE model).

Next to these three central predictors, we also consider five socio-demographic characteristics: gender, age, education, social capital and religious involvement. For most of these variables there are no apparent reasons to anticipate differential effects, and we consequently hypothesize that they are uniformly related to anti-Walloon and anti-immigrant attitudes. We expect men (H6), older people (H7), lower educated individuals (H8) and persons with low social capital (H9) to be more negative toward Walloons as well as immigrants (Zick, Küpper & Hövermann, 2011). The mechanism behind the impact of social capital is that being member of organisations and being integrated in community life fosters democratic values such as equality and pluralism, and therefore buffers prejudice in general (Putnam, 2000). Finally, religious involvement often goes hand in hand with higher levels of prejudice toward out-groups that are perceived as violating the religion’s value system (Hunsberger & Jackson, 2005). Because immigrants (of Turkish and Moroccan origin) are predominantly Muslims, while Flemings and Walloons share a Catholic background, we expect that religiosity is more strongly related to anti-immigrant prejudice than towards anti-Walloon prejudice (H10).

In line with Agnew, Thompson and Gaines (2000) and Allport’s lens model of prejudice (1954; Stephan, 2008), we construct a causal model were more distal factors of prejudice (i.e. structural predictors such as the socio-demographic variables described above) are mediated by more proximal factors of prejudice (i.e. attitudinal predictors). Studies explicitly investigating these kind of mediation models have indeed confirmed that attitudinal variables such as RWA, relative deprivation and national identity mediate the relationship between social structure and different types of prejudice (Carvacho et al., 2013; Hodson & Busseri, 2012; Pettigrew et al., 2008).

## Data and Methods

### Dataset

To test the hypotheses, we use the Flemish dataset of the Belgian National Election Study (BNES) 2010 organized by ISPO-KU Leuven (Swyngedouw & Abts, 2011). Between October 2010 and February 2011, a two-stage random probability sample of the Flemish residents was approached by means of a computer assisted personal interview conducted in Dutch with an average duration of 65 minutes. A sample of 711 persons older than 18 years and entitled to vote was realized (response rate: 65%). Because this study focuses on the structure of prejudices among majority-group members, respondents of immigrant background (operationalized as not having Belgian nationality or being born abroad) are excluded from the analysis, resulting in an effective sample size of 661. A small number of respondents (namely 3 out of 46 sampled clusters) live in the Brussels periphery. Due to privacy protection regulations the postal code has not been included in the data set. As a result, this small number of respondents could not be excluded or analysed separately.

### Indicators

*Dependent variables: anti-Walloon and anti-immigrant attitudes* – To enhance the comparison between anti-Walloon/Francophone and anti-immigrant attitudes as much as possible, we designed a very similar instrument for measuring attitudes towards both out-groups. Point of departure is a validated scale for negative attitudes towards (Turkish and Moroccan) immigrants that has been included in the BNES since the early 1990s (Billiet, 1995), consisting of 5-point Likert-type items referring to perceptions of symbolic threat, abuse of social security, general distrust of the out-group, negative stereotyping of immigrants as lazy, and preferential treatment of the out-group by the government over the in-group (Cronbach’s alpha: 0.88). The six items were reformulated to measure anti-Walloon/Francophone attitudes by replacing ‘immigrants’ by a reference to the Walloons (4 items) or Francophone Belgians (2 items). Apart from the change of mentioned target group, the wording of the items was kept as similar as possible (Cronbach’s alpha: 0.77; see Table 1 for the precise question wording). In the questionnaire, the battery on Walloons/Francophones was placed roughly 30 minutes after the anti-immigrant instrument to minimize the risk of bias resulting from memory effects.

**Table 1:** Frequency distributions for the anti-immigrant and anti-Walloon items.

			Disagree completely	Disagree	Neither agree, nor disagree	Agree	Agree completely	Mean	N

*Anti-Walloon attitudes*	q113_1	In general, Walloons cannot be trusted.	10.4	59.4	20.4	9.1	0.8	2.30	655
	q113_2	The Walloons take advantage of our social security system.	2.7	19.9	26.9	45.2	5.4	3.31	651
	q113_3	The Francophones are a threat to our culture and customs in the Brussels periphery.	2.7	27.8	28.7	35.8	5.1	3.13	645
	q113_4	The presence of Francophone culture enriches our society.	1.5	17.9	37.4	40.2	3.0	3.25	651
	q113_5	Most Walloons are lazy. They try to avoid exhausting and heavy work.	5.9	41.1	31.2	19.8	2.0	2.71	651
	q113_8	The government does more for the Walloons than for the Flemings.	1.8	31.7	33.4	28.4	4.6	3.02	637

*Anti-immigrant attitudes*	q68_1	In general, migrants cannot be trusted.	6.2	32.9	31.0	24.3	5.6	2.90	652
	q68_3	Migrants come here to take advantage of our social security system.	2.3	11.2	25.1	42.4	19.0	3.65	655
	q68_4	Migrants are a threat to our culture and customs.	3.7	26.7	17.9	40.4	11.4	3.29	656
	q68_5	The presence of different cultures enriches our society.	4.5	26.4	25.6	39.8	3.7	3.12	652
	q68_8	Most migrants are lazy. They try to avoid exhausting and heavy work.	5.0	35.9	31.3	20.7	7.2	2.89	651
	q70_4	The government does more for immigrants than for Belgians.	5.8	25.6	21.3	32.5	14.9	3.25	648

*Source*: Belgian National Election Study (BNES) 2010. Results are weighted for gender, age and education.

*Explanatory variables: Authoritarianism* is measured by means of three Likert-type items gauging the importance respondents place on obedience and respect (q81_5), strict laws (q81_6) and the punishment of immoral persons (q81_4) (Cronbach’s alpha: 0.59). To measure *group relative deprivation*, three items (q70_1–q70_3) referring to the feeling that the own group is being disadvantaged compared to other groups by the government and in times of economic crisis (Cronbach’s alpha: 0.84). *National vs. subnational self-identification* is operationalized by means of two items. The so-called Moreno question (Moreno, 2006) asks respondents to position themselves on a 5-point continuum ranging from exclusively Flemish to exclusively Belgian. A second item measures respondents’ opinions towards the distribution of political powers on an 11-point continuum ranging from ‘Flanders should decide – 0’ to ‘Belgium should decide – 10’ (*r* = 0.42). The structural equation models presented below indicate that these scales are sufficiently valid and reliable measurements for the intended concepts (see Appendix A1 for factor loadings).

Besides these key explanatory variables, various indicators of *social-structural position* are included, namely gender (a dummy variable with value 1 for women and 0 for men), age (in years), educational level (up to lower secondary degree – higher secondary degree – tertiary degree), religious involvement and social capital. For religious involvement, we make a distinction between Christians (mostly Catholics) who attend religious services on a regular basis (i.e. at least once per month); Christians who do not or only occasionally attend services; non-believers and free-thinkers. Social capital is measured by asking respondents whether they are member of organizations (such as sports clubs, socio-cultural organizations, neighbourhood committees, or voluntary associations). A distinction is made between respondents who are not member of any organization, and those who are member of at least one organization.

Descriptive statistics for the background variables are included in Appendix A2, and Appendix A3 displays the correlation matrix for the manifest variables that are used as predictors in the analysis.

### Statistical modelling

The statistical analysis is carried out in two major steps. In a first step, we focus on the confirmatory factor analysis (CFA) models for anti-Walloon and anti-immigrant attitudes. Responses on each of the twelve items (namely six per latent construct) are modelled as a function of the latent variable they load on:

1 \begin{array}{c}
{y_{ijk}} = {\tau _{ij}} + {\lambda _{ij}}{\eta _j} + {\varepsilon _{ijk}}\\
\ \ \ \ \ \ \ \ \ \ \ \ {\rm{with}}\ i = 1 \ldots 6; j = 1, 2; k = 1 \ldots \rm{N}
\end{array}

In expression (1), *y_ijk_* refers to the response of respondent *k* on item *i* measuring latent variable *j*. Hereby, *τ_ij_* refers to the item intercept, *λ_ij_* to the factor loadings, *η_j_* to the latent variable and *ɛ_ijk_* to the error term. In order to enhance the comparison of the two latent variables, we make use of the literature on measurement equivalence (for a review, see Davidov et al., 2014). This literature argues that valid comparisons require that measurement instruments are sufficiently invariant, and prescribes to test the equality of factor structures (configural equivalence; this guarantees that construct bias is absent), factor loadings (metric equivalence; this makes valid comparisons of regression effects possible) and/or of item intercepts (scalar equivalence; i.e. a requirement for latent mean comparisons) (Steenkamp & Baumgartner, 1998). Usually, this framework is applied to assess whether a measurement scale functions equivalently among different categories of respondents. In this study, the situation is quite different since we intend to compare two different measurement scales with matched items (namely anti-immigrant and anti-Walloon attitudes) for a single group of respondents. Nevertheless, the principles and levels of measurement equivalence can be applied to this situation as well. Concretely, rather than estimating a multi-group CFA (as is customary in equivalence testing) we will estimate a single-group CFA with two latent variables, and make comparisons of measurement parameters across the concepts. First, we will test configural equivalence by assessing whether both instruments exhibit a similar factor structure of salient and non-salient loadings. Second, we will assess whether the loadings for the matched items are identical (metric equivalence):

2 {\lambda _{i1}} = {\lambda _{i2}}\,\,{\rm{for}}\,\,\forall i \in \left\{{1 \ldots 6} \right\}

Third, the invariance of item intercepts will be evaluated (scalar equivalence):

3 {\tau _{i1}} = {\tau _{i2}}\,\,{\rm{for}}\,\,\forall i \in \left\{{1 \ldots 6} \right\}

**Figure 1:**
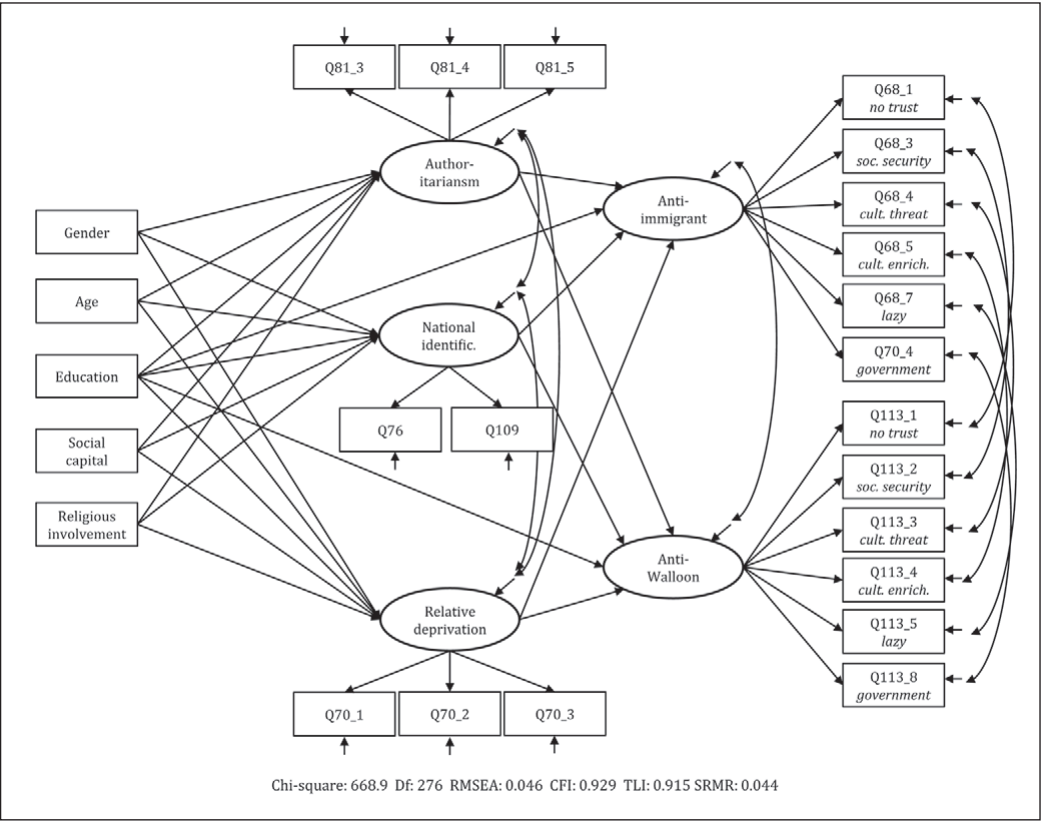
Explanation model for anti-Walloon and anti-immigration attitudes.

Once the measurement instruments (and their comparability) have been validated, we focus on the determinants of anti-Walloon and anti-immigrant attitudes in a second step. To this purpose, authoritarianism, relative deprivation, (sub)national identity and the social-structural variables are added to the model. The effect of the socio-economic background is mediated by authoritarianism, relative deprivation, and (sub)national identification. Direct effects of social background variables on the two prejudice types are only included when necessary (see Figure 1 for a graphical representation of this model). We use chi-square difference tests to investigate whether the determinants have an equal impact on anti-Walloon and anti-immigrant attitudes.

All reported models are estimated using Mplus version 7.1 (Muthén & Muthén, 1998–2012), using the default Full Information Maximum Likelihood (ML) estimator.^2^ The model fit is evaluated by assessing the Root Mean Squared Error of Approximation (RMSEA; should be below .06), the Comparative Fit Index (CFI) and the Tucker-Lewis Index (TLI; closer to 1 is better), and the Standardized Root Mean Residual (SRMR; expected to be lower than 0.08) (Hu & Bentler, 1999).

## Results

### Structure and prevalence of anti-Walloon and anti-immigrant attitudes

Before turning to the measurement models, we inspect the frequency distributions of the items measuring anti-Walloon and anti-immigrant attitudes. Table 1 shows that anti-Walloon opinions are prevalent among a sizeable group of the Flemish citizens. As expected, competition for the resources of the welfare state is found to be the most widespread source of perceived threat. More than 50% of the Flemish voters (strongly) agree that “*the Walloons take advantage of our social security system*”. Cultural threat perceptions are less outspoken: only 19% disagrees with the statement that Francophone culture is an enrichment (and thus no threat) for society. However, when the Brussels periphery is mentioned, the perceived Francophone threat increases to 41%. The finding that cultural threat perceptions are less pronounced than fear for competition over welfare resources is consistent with the observation that, apart from the Brussels region, Flemings and Francophones occupy separate cultural spaces but share a single socio-economic space in terms of the federal welfare state. Regarding the statement that the Belgian government treats Walloons preferentially over Flemings, the Flemish public opinion is divided. Almost equal shares of the population (strongly) agree with this statement, are in (strong) disagreement, or have no outspoken opinion on the matter. Finally, regarding negative stereotypes about Walloons, only 10% perceives Walloons as not trustworthy, while 22% endorses the statement that Walloons are lazy.

The frequency distributions for the anti-immigrant items generally show that Flemings harbour more negative attitudes towards immigrants than towards Walloons, but at the same time the differences are not overwhelming. Mentioning immigrants instead of Walloons typically increases agreement with negatively worded items by 10 percentage points. Similar as for anti-Walloon prejudice, social security threat seems to be the most salient source of anti-immigrant prejudice with 62% expressing fear that immigrants take advantage of the welfare state. Immigrants also elicit stronger cultural threat perceptions than Walloons: 52% sees immigrants as a “*threat to our culture and customs*”, while only 31% believes that cultural diversity enriches society. The differences between attitudes towards immigrants and Walloons are more distinct for the items on distrust and treatment by the government. Respectively 30% and 47% of the respondents (strongly) agree with the statement that immigrants are generally not trustworthy and are given preferential treatment respectively, which is markedly higher than similar evaluations of Walloons (10% and 33% respectively). Concerning the stereotype of lacking work ethic, finally, both target groups are relatively close in the eye of the Flemish citizen: 28% feels that immigrants are lazy.

Although these frequency distributions are instructive, a more in-depth analysis of the differences in prevalence and structure of anti-Walloon and anti-immigration attitudes requires a latent variable approach. Table 2 presents the fit indices for a series of nested CFA models implying various degrees of measurement invariance. Model 1 contains a single latent factor on which all the anti-Walloon and the anti-immigrant items load. This model does not provide an adequate description of the observed data: RMSEA is considerably larger than 0.06, and CFI and TLI are below 0.90. Clearly, Flemish attitudes towards Walloons and immigrants are not reducible to a single dimension.

**Table 2:** Fit indices for the measurement models of anti-immigrant and anti-Walloon attitudes.

		Chi2	Df	RMSEA	CFI	TLI	SRMR

M1	1 factor	535.8	54	0.116	0.846	0.811	0.073
M2	2 factors – no error correlations	171.3	53	0.058	0.962	0.953	0.035
M3	2 factors + error corr. – Equal configuration	98.5	47	0.041	0.983	0.977	0.027
M4a	2 factors + error corr. – Equal factor loadings	139.7	52	0.051	0.972	0.964	0.057
M4b	2 factors + error corr. – Partially equal factor loadings	117.0	50	0.045	0.979	0.972	0.042
M5a	2 factors + error corr. – Equal intercepts	224.0	53	0.070	0.945	0.932	0.050
M5b	2 factors + error corr. – Partially equal intercepts	125.3	52	0.046	0.976	0.970	0.045

*Source*: Belgian National Election Study (BNES) 2010; N = 661.

Specifying a separate anti-Walloon and anti-immigrant factor improves model fit dramatically (Model 2; ΔChi^2^ = 364.4; ΔDf = 1; *p* < .0001).^3^ Based on the modification indices, we added error covariances between the matched items to this two-factor model to take the similarity in question wording into account (see Figure 1), resulting in a satisfactory model fit (see Model 3 in Table 2). Apart from the two items measuring perceptions of being threatened by Francophone culture (see below for further explanation), all standardized factor loadings are larger than .60, which evidences that the items are sufficiently reliable and valid indicators of anti-Walloon and anti-immigrant attitudes.

We enhance the comparison of anti-Walloon and anti-immigration attitudes further by evaluating to what extent the measurement parameters are equal for both instruments. In a first step, we test whether item pairs have equal factor loadings. This equality of factor loadings implies that the various indicators -each referring to specific sources of threat or stereotypes- are to the same extent related to the two forms of prejudice. In other words, it evaluates whether or not anti-Walloon and anti-immigrant attitudes are structured along the same lines. Pair-wise equality constraints on the factor loadings (see Model 4a) decrease model fit substantially. Compared to Model 3, the chi-square value has increased significantly (ΔChi^2^ = 41.2; ΔDf = 5; *p* < .001), and RMSEA, CFI and TLI have become substantially worse. The modification indices point out that the misfit is primarily located in the two item pairs referring to cultural threat (Q113_3 & Q68_4; Q113_4 & Q68_5). Freeing up these two problematic equality constraints (Model 4b) improves model fit again almost up to the level of the unconstrained model. In case of the anti-Walloon factor, the loadings are substantially smaller than for the anti-immigrant factor. This means that Flemings’ perceptions of being culturally threatened are less relevant for the development of anti-Walloon attitudes than for anti-immigrant sentiment, confirming hypothesis 1b (based on the DT model), and rejecting hypothesis 1a (based on the GFE approach).

Finally, we also impose equality constraints on the intercepts of the item pairs.^4^ Equality of intercepts means that, conditional on the mean of the latent factor, the two items have the same expected value and that the items are unbiased with respect to the target group mentioned. In other words, a respondent who holds the same level of prejudice against Walloons and immigrants is expected to give the same response to a particular item. Implementing pair-wise equality constraints on the intercepts leads to a sharp decrease in model fit (Model 5a), but again the misfit is highly concentrated. Removing the equality constraint on the item pair measuring intergroup trust (Q113_1 & Q68_1) eliminates the lions’ share of the misfit that was induced by setting intercepts equal (see Model 5b). The distrust item has a considerably lower intercept when the Walloons are mentioned instead of immigrants. Flemish respondents who otherwise hold similar levels of prejudice against both out-groups nevertheless express lower levels of distrust towards Walloons than towards immigrants. Even among persons with an anti-Walloon disposition, distrust towards Walloons is relatively low.

This final model (Model 5b) has a satisfactory fit. Compared to Model 3, Model 5b does have a significantly higher chi-square value (ΔChi^2^ = 26.8; ΔDf = 5; *p* < .001). According to prevailing guidelines (Cheung & Rensvold, 2002), however, the alternative fit indices of Model 5b are only marginally lower than those of Model 3, and one can conclude that the remaining constraints on factor loadings and intercepts are supported by the data. Although some differences in factor loadings and intercepts are detected, three item pairs have completely invariant measurement parameters. This partial scalar equivalence (Byrne, Shavelson & Muthén, 1989) guarantees valid comparisons of anti-Walloon and anti-immigration attitudes at the level of the latent variables, both in terms of levels (means) as well as relation to other variables.

The parameter estimates for Model 5b (presented in Table 3) reveal a nuanced picture about the similarities and differences between both forms of prejudice. On the one hand, although we find two separate factors, anti-Walloon and anti-immigrant attitudes correlate very strongly (0.65). Clearly, negative attitudes towards Walloons and immigrants are distinct but closely connected phenomena. On the other hand, the two latent variables show some marked differences. First, the anti-Walloon factor has a lower latent mean (–0.20) than the anti-immigrant factor (this mean is fixed at 0 for reasons of model identification, and thus serves as reference point). The finding that anti-Walloon prejudice is lower compared to anti-immigrant prejudice confirms hypothesis 2. This difference is not only statistically significant, but also substantively relevant – it equals roughly one third of a standard deviation. Second, also the amount of variation differs clearly between the latent constructs. The anti-immigrant factor has a larger variance than the anti-Walloon factor (0.50 vs. 0.30). This indicates that the Flemish stands are more divided concerning the contentious issue of the presence of immigrants, while there is a somewhat more agreement regarding the regional/linguistic cleavage.

**Table 3:** Measurement parameters for anti-immigrant and anti-Walloon attitudes (Model 5b).

		Factor loadings (standardized)				Item intercepts	
		*Factor1: Anti-immig.*		*Factor2: Anti-Wal.*			
		
		Par.Est.	SE	Par.Est.	SE	Par.Est.	SE

q68_1	In general, migrants cannot be trusted.	0.73	(0.02)	–		2.93	(0.08)
q68_3	Migrants come here to take advantage of our social security system.	0.86	(0.01)	–		3.50	(0.10)
q68_4	Migrants are a threat to our culture and customs.	0.80	(0.02)	–		2.96	(0.09)
q68_5	The presence of different cultures enriches our society.	–0.60	(0.03)	–		3.17	(0.10)
q68_8	Most migrants are lazy. They try to avoid exhausting and heavy work.	0.75	(0.02)	–		2.81	(0.08)
q70_4	The government does more for immigrants than for Belgians.	0.69	(0.02)	–		2.80	(0.08)
q113_1	In general, Walloons cannot be trusted.	–		0.68	(0.02)	3.03	(0.09)
q113_2	The Walloons take advantage of our social security system.	–		0.74	(0.02)	3.89	(0.10)
q113_3	The Francophones are a threat to our culture and customs in the Brussels periphery.	–		0.57	(0.03)	3.44	(0.10)
q113_4	The presence of Francophone culture enriches our society.	–		–0.35	(0.04)	3.77	(0.12)
q113_5	Most Walloons are lazy. They try to avoid exhausing and heavy work.	–		0.66	(0.02)	3.17	(0.09)
q113_8	The goverment does more for the Walloons than for the Flemish.	–		0.66	(0.02)	3.43	(0.10)
		**Variances**				**Latent means**	
		
		**Par.Est.**	**SE**			**Par.Est.**	**SE**

	Factor 1: anti-immigrant attitudes	0.50	(0.04)			0.00	(–)
	Factor 2: anti-Walloon attitudes	0.30	(0.03)			–0.20	(0.03)
	Correlation (Factor 1, Factor 2)	0.65	(0.03)				

*Source*: Belgian National Election Study (BNES) 2010; N = 661. The model contains error covariances between the matched item pairs.

### Determinants of anti-Walloon and anti-immigrant attitudes

To assess the (dis)similarity in determinants of anti-Walloon and anti-immigrant prejudice, we add three individual difference variables -authoritarianism, group-relative deprivation and national identification- to the final measurement model, as well as a number of social background variables (gender, age, education, social capital and religious involvement). Because authoritarian dispositions, feelings of relative deprivation, and national identification can be influenced by social background, we include indirect (mediation) effects of social background on prejudice. Direct effects of social background are included in the model only if this leads to a significant improvement of model fit. This is only the case for education (ΔChi^2^ = 18. 5; ΔDf = 4; *p* < .001).

The finding that our measurement instrument is partially equivalent across target groups allows us to make formal comparisons of the size of effects on the two forms of prejudice. For each of the variables directly related to the prejudices (i.e. authoritarianism, relative deprivation, national identification and education), we tested whether there is a differential impact on anti-Walloon and anti-immigrant attitudes. Concretely, we compared models with and without equality constraints on the effects by means of chi-square difference tests. Constraining the direct effect of education does not deteriorate model fit significantly (ΔChi^2^ = 0.4; ΔDf = 2; *p* = .402). Authoritarianism (ΔChi^2^ = 10.1; ΔDf = 1; *p* < .001), relative deprivation (ΔChi^2^ = 14.0; ΔDf = 1; *p* < .001) and especially national identification (ΔChi^2^ = 44.7; ΔDf = 1; *p* < .001) do have a differential impact on both forms of prejudice. The resulting model (depicted in Figure 1) has a good model fit.

Table 4 gives the direct as well as indirect effects of the predictors on anti-Walloon and anti-immigrant attitudes, and presents the effects of the background variables on authoritarianism and relative deprivation. All continuous variables (but not the dummies for gender, education, religious involvement and social capital) are standardized. As a result, effects of continuous variables are standardized beta’s, while the effects parameters for dummies are semi-standardized (i.e. standardized for the dependent variable but not for the predictor) and thus refer to the number of standard deviations that a particular category deviates from the reference group.

**Table 4:** Full structural equation model explaining anti-immigrant and anti-Walloon attitudes.

	Relative deprivation	Authoritarianism	Sub-national identification	Anti-immigrant attitudes		Anti-Walloon attitudes	
	
				*Direct effect*	*Indirect effect*	*Direct effect*	*Indirect effect*

Gender							
*Male (ref.cat.)*							
*Female*	0.252***	0.140	–0.153		0.151*		0.040
Age (in years)	0.025	0.299***	–0.025		0.157***		0.115**
Educational level							
*Primary or lower secondary*	0.894***	0.816***	–0.430**	0.200*	0.710***	0.262*	0.333**
*Higher secondary*	0.556***	0.678***	–0.081	–0.073	0.548***	–0.095	0.378***
*Tertiary (ref.cat.)*							
Religious involvement							
*Non-believer/free-thinker*	–0.054	–0.300**	–0.136		–0.187*		–0.211*
*Occasional church attendance (ref.cat.)*							
*Regular church attendance*	–0.206*	–0.086	0.171		–0.104		0.004
Social capital							
*Not a member of association*	0.356***	0.175*	0.075		0.235***		0.205**
*Member of association (ref.cat.)*							
Relative deprivation				0.390***		0.261***	
Authoritarianism				0.502***		0.409***	
National identification (Flemish vs. Belgian)				0.113**		0.544***	
Proportion of explained variance	0.235	0.328	0.047	0.689		0.706	
Fit indices	Chi-square: 668.9*** Df: 276 RMSEA: 0.046 CFI: 0.929 TLI: 0.915 SRMR: 0.044						

*Source*: Belgian National Election Study (BNES) 2010; N = 661; * *p* < .05 ** *p* < .01 *** *p* < .001. Regression parameters are standardized or, in the case of dummy predictors semi-standardized (i.e. standardized for the dependent variable but not for the predictor). The impact of education is constrained to be equal across the two prejudices. Note that because the equality constraint is imposed on unstandardized parameters, standardized parameters can be different nevertheless.

Anti-Walloon and anti-immigrant attitudes are significantly related to each of the three individual difference variables. First, strong effects are found for authoritarianism. In line with expectations, authoritarian dispositions induce negative attitudes towards immigrants as well as Walloons/Francophones. Although the effect is very large for both forms of prejudice, authoritarianism has an even more outspoken impact on anti-immigrant attitudes than on anti-Walloon sentiments (0.502 vs. 0.409). This difference in effect size is in line with the idea that immigrants are, from the perspective of the Flemings, culturally more threatening than Walloons, and confirms hypothesis 4b. Second, sub-national identification with Flanders (instead of Belgium) increases prejudice. As predicted by the DT model (H3b), however, the negative impact of sub-national identification on anti-Walloon attitudes is considerably larger than that on anti-immigrant attitudes (0.544 vs. 0.113). In fact, sub-national identity is the strongest predictor of anti-Walloon attitudes in our model. This finding stems from the fact that Francophones and Walloons have served as the object of contra-identification for the definition of Flemish identity. Third, relative deprivation has a moderate influence on attitudes towards Walloons and immigrants. Flemings who feel unfavourably treated, compared to other social groups, report higher levels of prejudice against Walloons and immigrants. Although group relative deprivation contributes to attitudes towards both out-groups, it affects anti-immigrant attitudes to a slightly greater degree than anti-Walloon attitudes (thus rejecting hypothesis 5a). In sum, anti-Walloon and anti-immigrant are largely rooted in the same individual difference variables, but the effect sizes are different depending on the target of prejudice.

The effects of all background variables except education are fully mediated by authoritarianism and relative deprivation. Elderly persons hold more authoritarian worldviews and persons low on social capital score high on relative deprivation as well as authoritarianism. As a result, these social groups harbour more hostile attitudes towards immigrants as well as Walloons (which confirms H7 and H9). Females score higher on relative deprivation, which leads to slightly stronger anti-immigrant prejudices (thereby disproving H6). For educational level, the situation is somewhat more complex. The lower educated combine markedly higher levels of relative deprivation and authoritarianism (which leads to higher levels of prejudice in general) with weaker identification with the Flemish identity (which decreases especially anti-Walloon attitudes). As a result, we observe a strong negative indirect effect of education on anti-immigrant attitudes and a moderate negative indirect effect on anti-Walloon attitudes. In addition, education also has a small negative direct effect on both forms of prejudice. This means that, even taking their profile in terms of relative deprivation, authoritarianism and sub-national identification into account, the lower educated are slightly more prejudiced towards Walloons and immigrants than the higher educated. The total effect of education on both prejudices is outspokenly negative, which confirms hypothesis 8.

Regarding religious involvement, non-believers/free-thinkers hold less authoritarian dispositions compared to occasional church attendees (the reference category), leading to lower levels of anti-Walloon and anti-immigrant attitudes. Furthermore, regular church-goers feel less deprived than occasional church attendees. However, this difference is too small to affect prejudice indirectly and to produce the curve-linear effect reported in previous studies (Billiet, 1995; Hodson & Dhont, 2015). Contrary to hypothesis 10, religious background is not differentially related to anti-immigrant and anti-Walloon prejudices.

This analysis reveals that anti-immigrant and anti-Walloon attitudes are largely located within the same social strata. The reason is that the link between social structure and both forms of prejudice is largely and similarly mediated by relative deprivation, authoritarianism and to a lesser extent also sub-national identification. Overall, this model is very successful at explaining anti-immigrant and anti-Walloon attitudes: The proportions of explained variance equal respectively 0.69 and 0.71.

## Conclusion

This study sheds more light on the structure, the prevalence and the determinants of anti-Walloon attitudes by comparing this form of prejudice with anti-immigrant sentiments. For this purpose, we combined two theoretical perspectives. On the one hand, the Group-Focused Enmity (GFE) approach predicts that prejudices towards Walloons and immigrants are identically structured, and are similarly rooted in individual difference variables (such as authoritarianism, group relative deprivation and in-group identification). The Differentiated Threat (DT) model, on the other hand, postulates that both forms of prejudice are contingent on the respective contexts of intergroup relations and the types of threat involved, and therefore show different structures, levels and determinants. To test these propositions, we analyzed comparable instruments for anti-Walloon and anti-immigrant prejudice included in the Flemish dataset of the Belgian National Election Study (BNES) 2010.

Our results reveal a nuanced picture regarding the (dis)similarities in structure, level and roots of anti-Walloon and anti-immigrant attitudes in Flanders. On the one hand, and in line with GFE, the measurement models evidenced a high degree of correspondence between anti-Walloon and anti-immigration attitudes. In terms of internal structure, economic considerations –such as social security threats and perceptions of a lacking work ethic- seem to be a crucial ingredient for both forms of prejudice. In this sense, Walloons are, in the eye of the Flemish, indeed part of the “Band of Others” (Kalkan et al., 2009). On the other hand, besides these resemblances, we also detect context-specific differences in the structure of both types of prejudice that should not be overlooked. Compared to anti-immigrant attitudes, anti-Walloon attitudes are not only less outspoken among the Flemings, but have also a smaller variation and are less rooted in notions of cultural threat. These results illustrate that anti-Walloon and anti-immigrant attitudes are not completely structured along the same logics, as cultural frames are less important in the structuring of anti-Walloon prejudice. The patterns of predictors reveal analogies and differences as well. Both forms of prejudice are found to be largely located within the same social strata (i.e. elderly persons, the low educated and those low in social capital). Group-relative deprivation and authoritarianism tend to reinforce prejudice towards Walloons and immigrants, although these two variables have a significantly stronger effect on anti-immigrant attitudes. By contrast, anti-Walloon prejudice, is far more closely linked to feelings of identification with the Flemish in-group. This latter finding highlights that Francophones and Walloons have served -historically but also more recently- as the object of contra-identification for the construction of Flemish identity.

Besides providing insight into anti-Walloon attitudes, this study also contributes to scholarly discussions about the nature and genesis of prejudices. The empirical analysis evidences that the different dominant paradigms -what we called the GFE and the DT approach- should be treated as complementary rather than as contradictory approaches (for a similar argument, see Bergh & Akrami, 2016). The GFE approach rightfully stresses that specific prejudices have generic components that overlap with prejudices towards other out-groups. As a result, explanation models developed for generalized prejudice (such as authoritarian dispositions, feelings of deprivation or social identities) are a useful starting point to explore relatively unknown forms of prejudice, such as anti-Walloon attitudes. Yet, the DT model supplements these general insights by drawing the attention to the existence of specific components that are rooted in the historical-social-political context of intergroup relations. As such, insight in structural and contextual factors — such as patterns of social contact and competition, economic and power relations as well as media and elite discourses — is indispensable to fully understand why and how specific prejudices are triggered.

Finally, this study also makes a contribution to the methodological literature by illustrating how step-wise estimation of structural equation models can be used for comparing different forms of prejudice. To the best of our knowledge, this study is the first to apply the principles of (configural, metric and scalar) measurement equivalence on a comparison across matched measurement instruments (rather than groups of respondents). This approach has proven to be very useful in providing detailed insight into differences in (factor) structure, levels (i.e. latent means) and determinants of specific prejudices. This approach highlights the importance of assessing to what extent measurement scales are comparable before drawing substantive conclusions. Preferably, future research should replicate this approach on measurements including a wider variety of out-groups, so that a broader generalized prejudice factor can be constructed.

Some limitations of the study have to be acknowledged. First, the GFE and DT approach were only applied to two types of prejudice only. This is, of course, a rather narrow comparison base to allow a stringent test of both frameworks. Both prejudice types studied here are typically activated by similar kind of threats (although the cultural threat dimension is somewhat less relevant for attitudes towards Walloons/Francophones). As a result, this design might be biased towards the GFE argument. If more distinct out-groups, such as sexual minorities, were to be added to the comparison, it is likely that stronger differences would come to the surface and that more evidence for the DT approach would be found. Therefore, for now, the conclusions remain limited to two specific out-groups, but our theoretical and empirical model can be applied to more prejudice cases in different contextual settings. Second, our test neglects potential regional differences in threat perceptions within Flanders. In the Brussels periphery, where Flemish and Francophones share one cultural space, it is very well possible that cultural threats trump economic ones, and that the level, structure and roots of anti-Walloon/Francophone attitudes are quite distinct compared to the rest of Flanders. Due to characteristics of our sample, however, it was not possible to explore this issue.

## Additional File

The additional file for this article can be found as follows:

10.5334/pb.336.s1 Appendix 1 Tables.

## Footnotes

We prefer to use the term anti-Walloon rather than anti-Francophone because factor analysis shows that economic considerations are more central in this concept than cultural ones – see section 4.1.

Because SEM for ordered-categorical data complicates equivalence tests considerably, we decided to treat the Likert-type items as continuous data. This choice is legitimized by simulations indicating that ignoring the ordinal character of the data does not bias results, as long as the sample size is sufficiently large, the items contain at least 5 scale points and distributions are not overly skewed (DiStefano, 2002). Apart from the equivalence tests, we re-analysed the data using the WLSMV estimator for categorical data (results available upon request) and obtained very similar results.

Additionally, we also tested a CFA model that distinguishes between an anti-Walloon (items Q113_1, Q113_2, Q113_5 and Q11_8) and anti-Francophone (Q113_3 and Q113_4) attitudes. These two factors correlate almost perfectly (0.961), which illustrates that attitudes towards Walloons and Francophones are blurred in the Flemish public opinion, and that they cannot be analysed separately.

The intercepts for the two cultural item pairs are not set equal, because the factor loadings were already allowed to vary for these items. When factor loadings (slopes) are different, the equality of intercepts becomes meaningless.
