# Supplementary material for: Walloons as General or Specific Others? A Comparison of anti-Walloon and anti-immigrant Attitudes in Flanders
Source: Psychol Belg. 2017 Nov 21;57(3):75–97. doi: 10.5334/pb.336 (PMC6194540; doi:10.5334/pb.336)
Supplement: Appendix 1 — Tables. [file pb-57-3-336-s1.pdf]

Appendix

Table A1. Measurement model for authoritarianism, relative deprivation, and national identification

		Factor loadings (standardized)					
		Relative deprivation		*Authori-tarianism*		*National identification*	
		Par.Est.	SE	Par.Est.	SE	Par.Est.	SE
Q70_1	If we need something from the government, people like me have to wait longer than others	0.81	(0.02)	- -		- -	
Q70_2	People like me are being systematically neglected, whereas other groups receive more than they deserve.	0.87	(0.02)	- -		- -	
Q70_3	In times of economic crises people like me are always the first victims.	0.73	(0.02)	- -		- -	
Q81_4	Most of our social problems would be solved if we could somehow get rid of the immoral, crooked people.	- -		0.51	(0.04)	- -	
Q81_5	Obedience and respect for authority are the two most important virtues children have to learn.	- -		0.66	(0.03)	- -	
Q81_6	Laws should become stricter because too much freedom is not good for people.	- -		0.54	(0.04)	- -	
Q76	The form of state that the country should have is still a matter of discussion. Some think that "Flanders and Wallonia should each be able to decide over everything by themselves". Others think that "Belgium, Flemings and Walloons together, should be able to decide about everything". Where would you place yourself on the scale? (0 - Flanders should decide - 10: Belgium should decide)	- -		- -		-0.679	(0.04)
Q109	Some people feel primarily Flemish/Walloon, others primarily Belgian. Which of the following statements applies to you most? (1: Only Flemish - 5: Only Belgian)	- -		- -		-0.607	(0.04)

Source: Belgian National Election Study (BNES) 2010; Parameter estimates taken from model 5b (see Figure 1 and/or Table 2)

Table A2: Descriptive statistics of manifest independent variables

		Percentage	N
Gender	*Male*	54.01	357
	Female	45.99	304
	Total	100.00	661
Educational level	*Primary or lower secondary*	29.29	193
	Higher secondary	37.94	250
	Tertiariy	32.78	216
	Total	100.00	659
Religious involvement	*Non-believer/ free-thinker*	24.66	163
	Occasional church attendance	45.54	301
	Regular church attendance	29.80	197
	Total	100.00	661
Social capital	*Not a member of association*	44.48	294
	Member of association	55.52	367
	Total	100.00	661

	Mean	STD	Range	N
Age (in years)	50.79	18.26	18-90	661

Table A3: Correlations between manifest predictor variables

	Q70_1	*Q70_2*	*Q70_3*	*Q81_4*	*Q81_5*	*Q81_6*	*Q109*	*Q76*	*Gender: Female*	*Social capital*	*Age*	*Education: lower sec.*	*Education: higher sec.*	*Relig.: non-believer*	*Relig.: regular church.*
Q70_1	1.00														
Q70_2	0.71	1.00													
Q70_3	0.58	0.63	1.00												
Q81_4	0.18	0.26	0.20	1.00											
Q81_5	0.31	0.35	0.29	0.31	1.00										
Q81_6	0.20	0.19	0.16	0.27	0.40	1.00									
Q109	-0.09	-0.07	0.01	-0.06	0.01	-0.09	1.00								
Q76	-0.01	-0.02	0.06	-0.05	0.11	0.05	0.41	1.00							
Gender: Female	0.12	0.12	0.10	0.00	0.06	0.06	0.06	0.04	1.00						
Social capital: no member	0.23	0.24	0.28	0.10	0.15	0.12	-0.02	0.11	0.12	1.00					
Age	0.09	0.10	0.15	0.15	0.23	0.30	0.01	0.09	-0.01	0.15	1.00				
Education: lower sec.	0.25	0.24	0.34	0.17	0.26	0.14	0.06	0.18	0.01	0.25	0.35	1.00			
Education: higher sec.	0.06	0.07	0.02	0.05	0.07	0.03	-0.05	-0.04	-0.03	0.02	-0.17	-0.50	1.00		
Relig.: non-believer	-0.03	-0.05	-0.02	-0.06	-0.13	-0.13	0.06	-0.02	-0.11	-0.08	-0.16	-0.08	0.03	1.00	
Relig.: regular church.	-0.08	-0.08	-0.01	0.01	-0.01	0.12	-0.07	0.00	0.04	0.00	0.19	0.02	-0.07	-0.37	1.00
